# Early monocyte subset signatures predict outcomes in unvaccinated COVID-19 patients

**DOI:** 10.3389/fimmu.2026.1882388

**Published:** 2026-09-15

**Authors:** Fabrício Marcus Silva Oliveira, Mônica Maria Magalhães Caetano, Larissa Lilian de Oliveira, Juliana Vaz de Melo Mambrini, Marina Santos Rezende, Marina Pinheiro Rocha Fantini, Tiago Antônio De Oliveira Mendes, Henrique Cerqueira Guimarães, Jacqueline Araújo Fiuza, Soraya Torres Gaze

**Affiliations:** 1 Fundação Oswaldo Cruz, Fiocruz, Instituto René Rachou, Fiocruz Minas, Oswaldo Cruz Foundation, Fiocruz, Belo Horizonte, Minas Gerais, Brazil; 2 Risoleta Tolentino Neves Hospitalonte, Belo Horiz, Brazil; 3 ECMO Minas, Belo Horizonte, Minas Gerais, Brazil; 4 Materdei Hospital, Belo Horizonte, Minas Gerais, Brazil; 5 Department of Biochemistry and Molecular Biology, Federal University of Viçosa, Viçosa, Minas Gerais, Brazil; 6 Department of Pathology and Parasitology, Federal University of Alfenas, Alfenas, Minas Gerais, Brazil

**Keywords:** COVID-19, monocyte subpopulations, outcome, SARS-CoV-2, unvaccinated patients

## Abstract

**Introduction:**

Severe acute respiratory syndrome coronavirus 2 (SARS-CoV-2) infection is characterized by complex immune responses, with monocytes playing a central role in disease pathogenesis.

**Objective:**

This study aimed to evaluate the profiles of monocytes and their receptor expression during the first week of hospitalization in unvaccinated patients with COVID-19, stratified by clinical outcome: discharged or deceased.

**Methods:**

Peripheral mononuclear cells from hospitalized patients were analyzed by flow cytometry to characterize classical, intermediate, and non-classical monocytes, along with the expression of CD169, CD209, and TLR2, TLR3, TLR4, and TLR9.

**Result:**

Patients in whom death was the outcome exhibited a significant reduction in classical monocytes and an expansion of non-classical monocytes early during hospitalization. Additionally, non-classical monocytes from deceased patients showed increased expression of CD169 and greater intensity of CD209 expression. Enhanced expression of TLR2 across all monocyte subsets and increased TLR4 expression specifically in non-classical monocytes were also associated with mortality. Correlation network analysis revealed more complex and dysregulated interactions among monocyte subsets and immune markers in deceased patients compared with discharged individuals.

**Conclusion:**

Our findings demonstrate that, during the first week of hospitalization, alterations in monocyte distribution and activation status are strongly associated with COVID-19 severity. Increased expression of CD169, CD209, TLR2, and TLR4, particularly in non-classical monocytes, reflects heightened innate immune activation and may contribute to poorer disease outcomes. Collectively, these results highlight the potential of monocyte subsets and their associated receptors as early prognostic biomarkers and provide insight into the immunopathogenesis of severe COVID-19.

## Graphical Abstract

**Figure:**
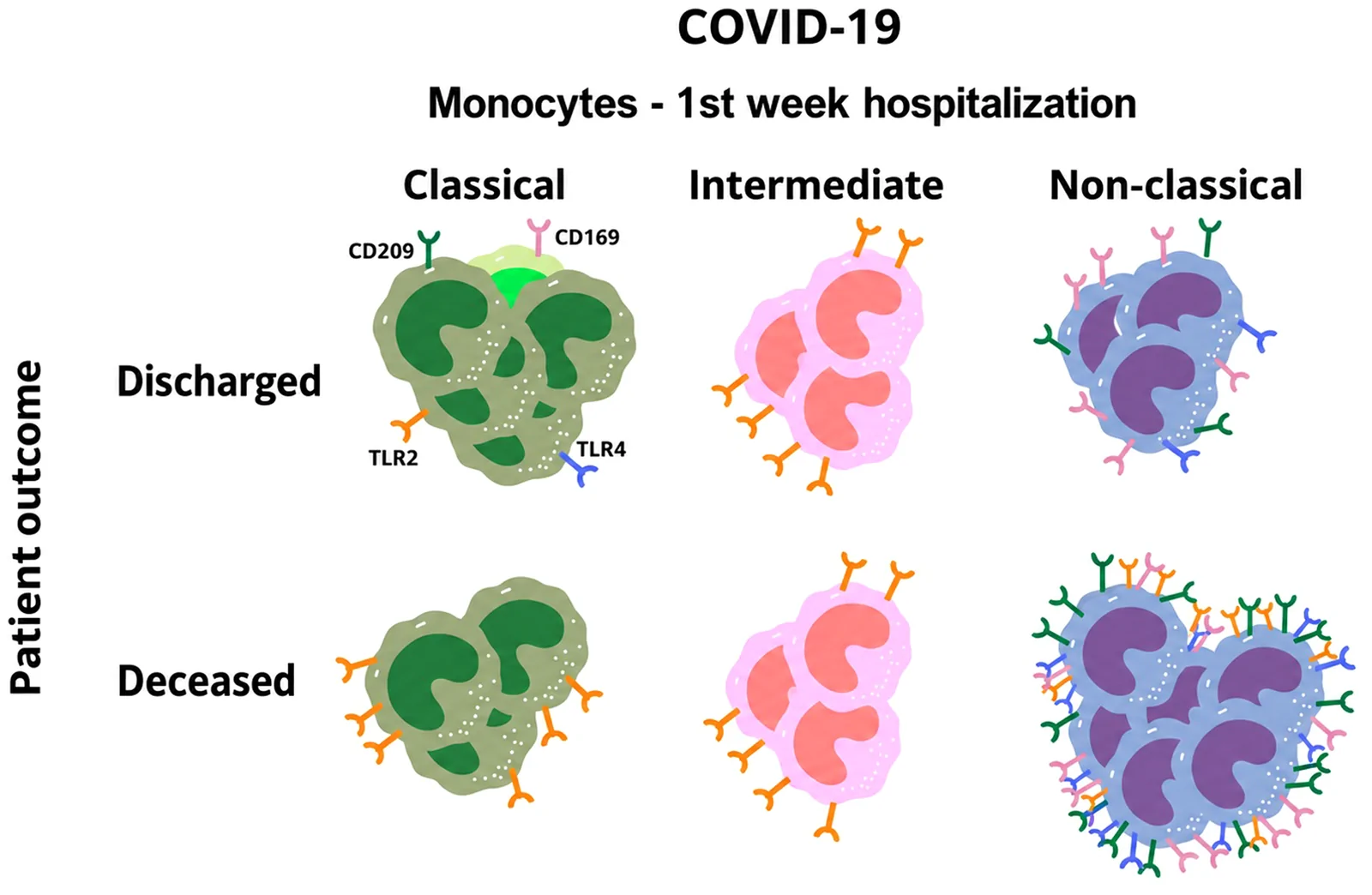

## Introduction

The severe acute respiratory syndrome coronavirus 2 (SARS-CoV-2) pandemic, which began in 2019/2020, brought new perspectives to immunological studies (1–3). During the course of the coronavirus disease 2019 (COVID-19) pandemic, the scientific world was actively investigating virus-cell interactions and developing new vaccines (4–7). Among immune cells, monocytes/macrophages have a pivotal role in the adaptive immune response, including the response to SARS-CoV-2 (8).

Monocytes circulate in the blood and can migrate to infected tissues upon recruitment. Upon tissue infiltration, they are able to differentiate into macrophages or dendritic cells, acquiring distinct functions that contribute to homeostasis, immune defense, and tissue repair (9, 10). They express several surface molecules, including CD14 and CD16, which are important for their subclassification into intermediate CD14^bright^CD16^+^ monocytes, classical CD14^bright^CD16^-^ monocytes, and non-classical CD14^bright^CD16^++^ monocytes (11–13). Monocytes expressing Siglec-1 (CD169) have been associated with acute SARS-CoV-2 infection. During the course of the disease, an increased percentage of these cells has been observed during hospitalization and has been associated with mortality (14, 15). Therefore, increased Siglec-1 (CD169) expression has been associated with COVID-19 severity.

Recently, the molecule DC-SIGN (dendritic cell-specific intercellular adhesion molecule-3-grabbing non-integrin), also known as CD209, has been reported to bind to and enhance SARS-CoV and SARS-CoV-2 infection independently of ACE2 expression (16–18). DC-SIGN (CD209) is a surface receptor expressed on dendritic cells (DCs) that interacts with the cell adhesion molecule ICAM-3 on T cells, thereby promoting DC–T lymphocyte interactions (19). This molecule is linked to increased levels of cytokines IL-4, IL-6, IL-10, and IL-13 during SARS-CoV-2 infection and can form multiple complexes with the spike protein, promoting viral entry into cells and trans-infection of T cells (18, 20). Given that monocytes have the capacity to differentiate into dendritic cells (10), evaluating the expression of CD209 in monocytes from COVID-19 patients represents an attractive approach to better understand their role in viral pathogenesis and their potential use as immunological markers of the disease (21).

Toll-like receptors (TLRs) constitute a family of pattern recognition receptors that detect a broad spectrum of pathogen-associated molecular patterns (PAMPs) (22). Monocytes express the full repertoire of TLRs, with TLR1, TLR2, and TLR4 being particularly abundant (23, 24). It has been demonstrated that TLR2, TLR4, and TLR9 are involved in the pathogenesis of COVID-19. For instance, prophylactic intranasal administration of a TLR2/6 agonist (INNA-051) significantly reduced SARS-CoV-2 viral RNA levels in the upper respiratory tract and decreased viral transmission in a ferret model (25). Moreover, it has been shown that TLR2 binds to the E protein, TLR4 to the spike protein, and TLR7 may bind to the S protein of SARS-CoV-2.Therefore, these TLRs may help facilitate viral entry into cells and maintain the infection (26–28). In addition, modulation of the TLR9 signaling pathway via mTOR inhibition has been proposed as a potential therapeutic strategy to reduce SARS-CoV-2-induced inflammation (29). On the other hand, TLR3 has been linked to a protective response to SARS-CoV and Middle East respiratory syndrome (MERS) infections, which are caused by viruses in the same family as SARS-CoV- 2 (30, 31).

In this study, we evaluated different monocyte subpopulations and their receptor expression during the first week of hospitalization for COVID-19 in unvaccinated patients stratified by outcome: discharged or deceased. We observed that deceased patients had a higher percentage of non-classical monocytes, with increased expression of CD169, CD209, TLR2, and TLR4 in this subpopulation. In addition, deceased patients exhibited a more complex network of interactions among these immune parameters. In summary, our findings demonstrate and emphasize that, even during the first week of hospitalization, patients already display differences in the blood monocyte compartment associated with disease severity, although the clinical outcome may occur several days after the time of assessment.

## Results

### Monocyte subpopulations are different between discharged and deceased COVID-19 patients during the first week of hospitalization

Monocytes and macrophages are described as important immune cell types in patients with COVID-19 and appear to play a central role in the pathogenicity of the disease (32), warranting attention and better characterization of their profile during the course of the disease. Our study aimed to contribute to characterizing immunophenotyped monocytes as prognostic markers during the acute phase of SARS-CoV-2 infection. We initially characterized these monocyte subsets during the first week of hospitalization, comparing patients who were discharged with those who died. Blood monocytes were evaluated according to their expression of CD14 and CD16 using the strategy shown in **Supplementary Figure 1**. Monocyte subpopulations were divided into classical (CD14^bright^CD16^-^, **Figure 1A**), intermediate (CD14^bright^CD16^+^, **Figure 1B**), and non-classical (CD14^-^CD16^+^, **Figure 1C**) subpopulations. Patients with the worst outcome, that is, those who died, had a lower percentage of classical monocytes and a higher percentage of non-classical monocytes (**Figures 1A, C**).

**Figure 1:**
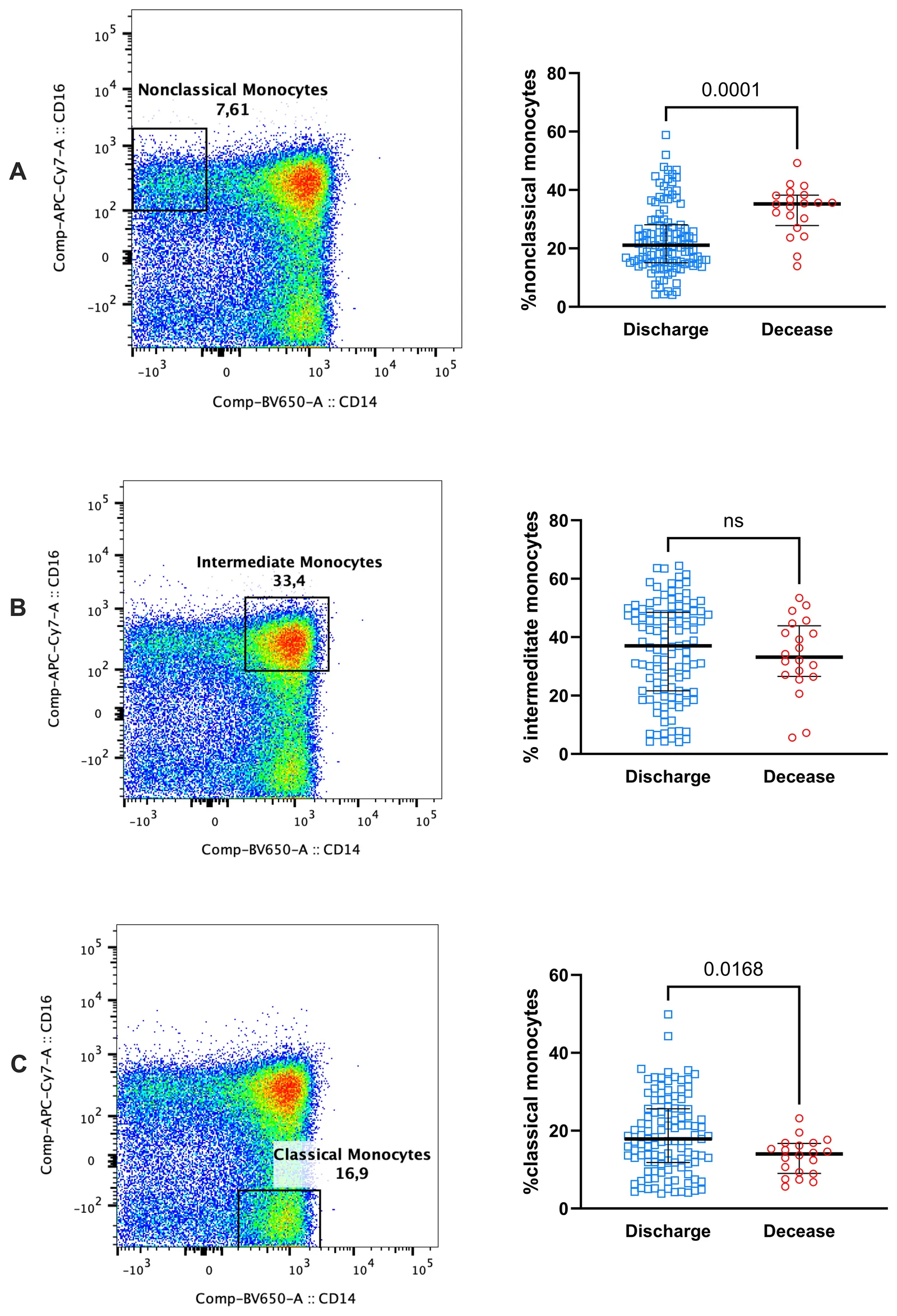
Distribution of monocyte subsets in COVID-19 patients according to clinical outcome. Peripheral blood monocytes from hospitalized COVID-19 patients were immunophenotyped during the first week of infection and stratified according to clinical outcome (discharged versus deceased). Monocyte subsets were defined based on CD14 and CD16 expression as classical [CD14^bright^CD16^−^; **(A)**], intermediate [CD14^bright^CD16^+^; **(B)**], and non-classical [CD14^−^CD16^+^; **(C)**]. The statistical values are presented in the graph.

### Deceased patients had higher expression of CD169 and CD209 in non-classical monocytes

Specific subtypes of monocytes expressing CD169 have been associated with the severity of SARS-CoV-2 infection and have been proposed as promising immunological biomarkers (14). We previously demonstrated that non-classical monocytes were more abundant in deceased patients compared with discharged patients during the first week of hospitalization (**Figures 1A–C**). Our next step was to characterize the expression of CD169 in monocyte populations, along with the expression of CD209, a protein associated with increased production of pro-inflammatory cytokines during SARS-CoV-2 infection (20). Overall, monocytes from deceased patients exhibited higher expression of CD209 and CD169 compared with those from discharged patients (**Figures 2A–G**). CD169 was more frequently expressed in non-classical monocytes in deceased patients than in discharged patients (**Figures 2A, B**). Moreover, this population also expressed higher levels of these receptors per cell during week 1 of hospitalization (**Figure 2C**). It is important to mention that this molecule has been linked to severity and death in COVID-19 patients, but this is the first time it has been demonstrated to be expressed in a non-classical monocyte subpopulation during week 1 of hospitalization. On the other hand, there was no difference in the percentage of monocytes expressing CD209 (**Supplementary Figures 2G–I**). Nevertheless, the intensity of expression was higher in intermediate and non-classical monocytes during week 1 of hospitalization in deceased patients, demonstrating its importance during the disease and its association with a worse outcome (**Figures 2D–G**).

**Figure 2:**
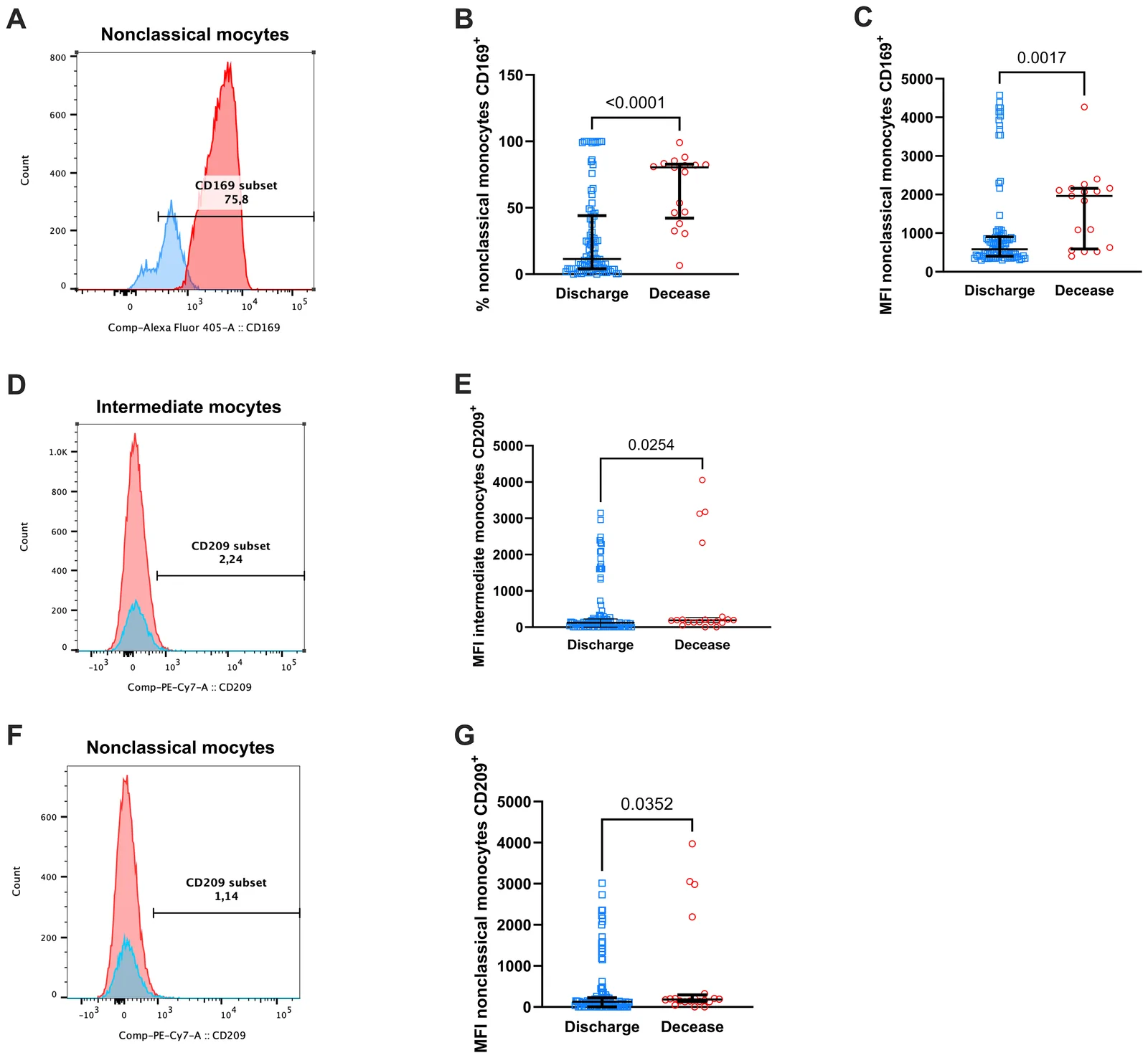
CD169 and CD209 expression by monocyte subsets. Peripheral blood monocytes from hospitalized COVID-19 patients were analyzed during the first week of hospitalization to assess the expression of CD169 and CD209 in deceased and discharged patients. Expression of CD169 by non-classical monocytes is represented by a histogram **(A)**, a percentage **(B)**, and a median fluorescence intensity (MFI) **(C)**. Expression of CD209 by intermediate monocytes is represented by a histogram **(D)** and MFI **(E)**. Expression of CD209 by non-classical monocytes is represented by a histogram **(F)** and an MFI **(G)**. The statistical values are presented in the graph.

Taken together, these findings indicate that non-classical monocytes from deceased patients exhibit increased expression of CD169 and CD209 during the first week of hospitalization, suggesting their potential use as immunological markers associated with severe SARS-CoV-2 infection.

### TLR2- and TLR4-expressing monocytes are associated with worse outcomes in COVID-19 patients during week 1 of hospitalization

Previous studies have demonstrated, through various mechanistic pathways, that Toll-like receptors are involved in the pathogenesis of COVID-19, highlighting a field that remains to be further explored to better our understanding of the infection (25, 30). In this study, we evaluated the expression of TLRs across different monocyte subsets.

During week 1 of hospitalization, all monocyte subpopulations, intermediate, classical, and non-classical, expressed more TLR-2 in deceased COVID-19 patients than in discharged patients (**Figures 3A–F**). However, the intensity of TLR2 expression was not different in those populations between the two studied groups (**Supplementary Figures 3D–F**). When evaluating TLR4, only non-classical monocytes showed a higher percentage and expression of this molecule in deceased patients compared with discharged patients during week 1 of hospitalization (**Figures 3G–I**), demonstrating the importance of this monocyte population in the severity of COVID-19. TLR3 and TLR9 were also evaluated in monocyte subpopulations. However, there was no significant difference between discharged and deceased groups (data not shown). These results highlight the potential of dysregulated TLR2 and TLR4 expression as immune markers of COVID-19 severity.

**Figure 3:**
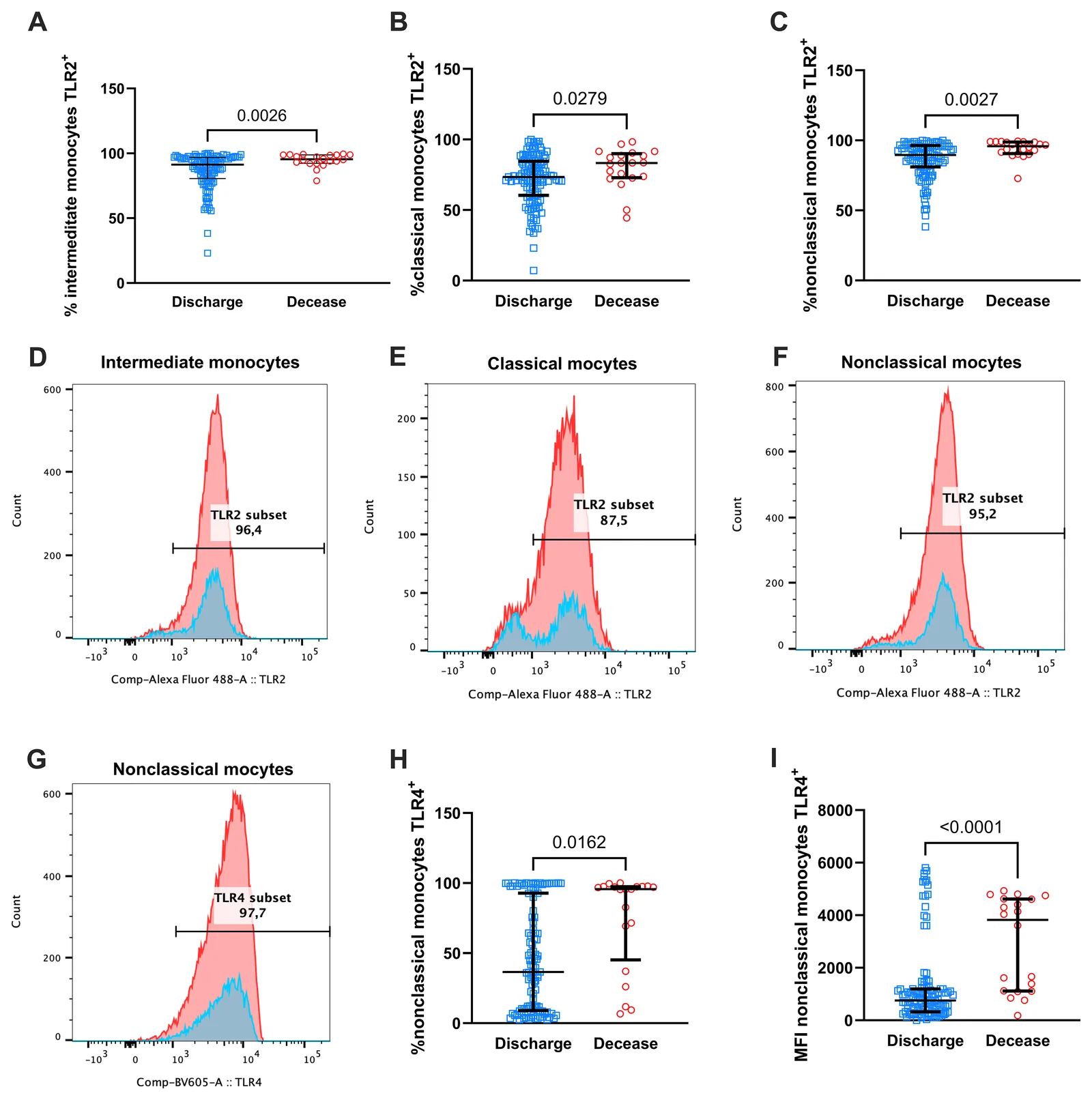
TLR2 and TLR4 expression by monocyte subsets. Peripheral blood monocytes from hospitalized COVID-19 patients were analyzed during the first week of hospitalization to assess the expression of CD169 and CD209 in deceased and discharged patients. The percentage of TLR2 expression in intermediate **(A)**, classical **(B)**, and non-classical monocytes **(C)**. Histograms representing TLR2 expression **(D–F)**. The expression of TLR4 by non-classical monocytes is represented by a histogram **(G)**, a percentage **(H)**, and an MFI **(I)**. The statistical values are presented in the graph.

### Correlation networks show important differences in monocyte relationships during the first week of hospitalization in COVID-19 patients

Finally, we investigated the correlation networks between monocyte subsets and the expression of their respective markers during the first week of hospitalization in COVID-19 patients stratified by clinical outcome (discharge or death). Correlation network analyses were performed using only strong values (r > 0.7 for positive correlation or r <-0.7 for negative correlation). In discharged patients, correlations among monocyte subpopulations involved the same receptor (**Figure 4A**). In general, the percentage of intermediate monocytes increased with the percentage of non-classical or classical monocytes expressing the evaluated molecule. In contrast, the relationship between monocyte subpopulations in deceased patients showed more complex interactions (**Figure 4B**), in which monocytes expressing different molecules were associated with increases in other monocyte populations (**Figure 3C**). Notably, only deceased patients showed a negative correlation between the percentage of intermediate monocytes and the percentage of non-classical monocytes expressing CD169, which may influence the outcome pathway. The main correlations observed in the heatmap were organized into correlation networks to facilitate visualization.

**Figure 4:**
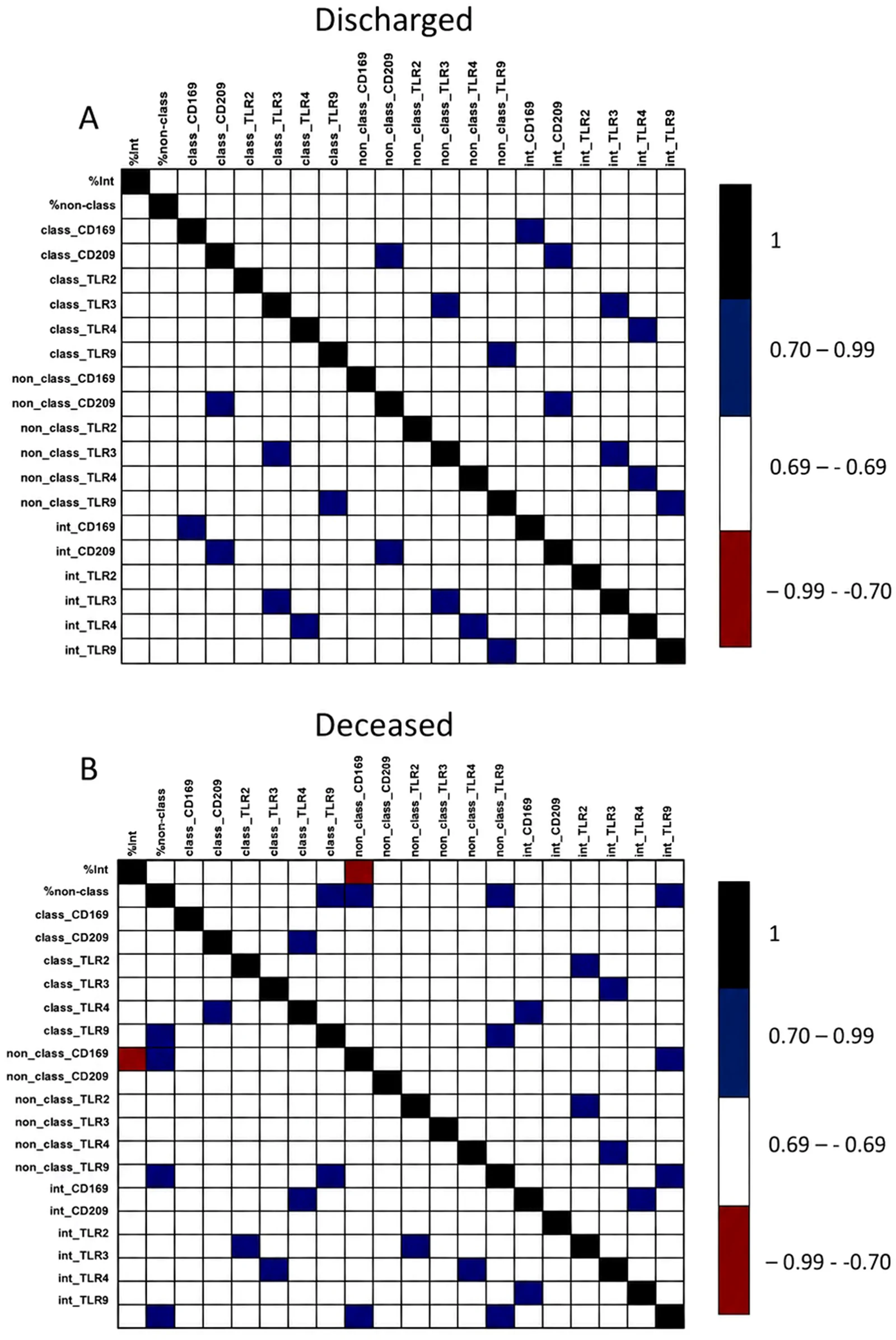
Correlation of monocyte subsets and marker expression according to clinical outcome in COVID-19 patients.Correlations were constructed to evaluate the relationships between monocyte subsets and the expression of associated markers during the first week of hospitalization in COVID-19 patients, stratified by clinical outcome (**A**-discharged vs. **B**-deceased). Only strong correlations were included in the analysis (r>0.7 for positive correlations and r<−0.7 for negative correlations).

To summarize the correlation, networks were performed (**Figure 5**). There were some interactions inherent to the first week of hospitalization in COVID-19 patients. Here, it is important to observe that intermediate monocytes expressing TLR3 increased together with classical monocytes expressing the same molecule. In addition, the expression of TLR9 in non-classical monocytes correlated with the expression of this molecule in the other two monocyte subpopulations (**Figure 5A**). During the first week of hospitalization, discharged patients showed that intermediate monocytes expressing one molecule increased with the other two monocyte subpopulations expressing the same molecule. This was observed for CD209 and TLR4. Interestingly, intermediate monocytes expressing CD169 increased with classical monocytes expressing CD169, and intermediate monocytes expressing TLR3 increased with non-classical monocytes expressing TLR3. The latter population then increased with classical monocytes expressing TLR3 (**Figure 5B**). These interactions probably reflect the maturation of intermediate monocytes into classical or non-classical monocytes expressing the same molecule, which is important for a positive prognosis in patients with COVID-19.

**Figure 5:**
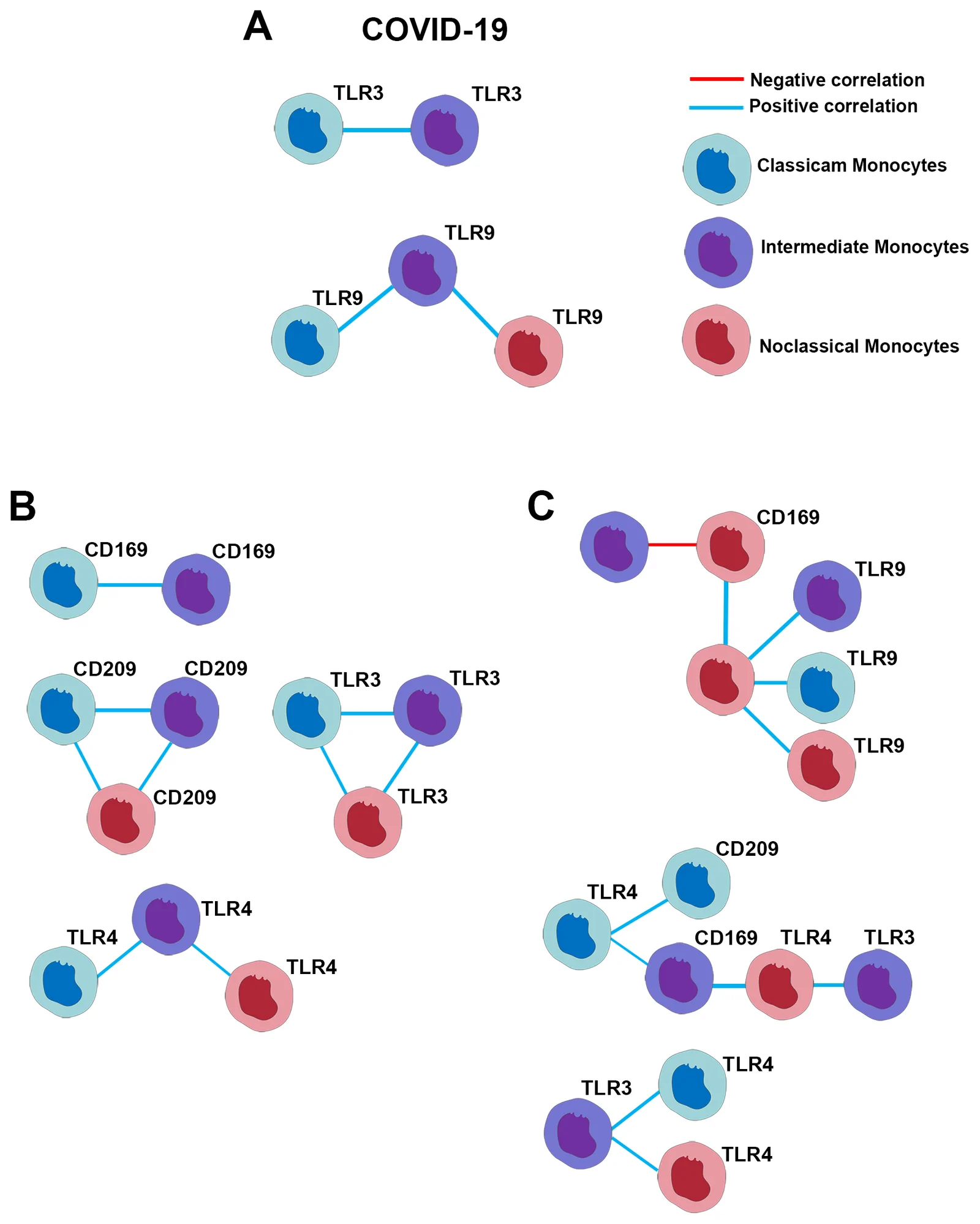
Correlation networks during the first week of hospitalization in COVID-19 patients. Correlation networks were generated to identify interactions between monocyte subsets and associated markers during the first week of hospitalization in COVID-19 patients. **(A)** Correlation networks present in discharged and deceased patients. **(B)** Correlation networks exclusively present in discharged patients. **(C)** Correlation networks exclusively present in deceased patients.

The network interaction during week 1 of hospitalization in deceased patients was more complex compared with that in discharged patients (**Figure 5C**). Exclusively in deceased patients, one negative correlation was observed. The percentage of intermediate monocytes in these patients decreased with the percentage of non-classical monocytes expressing CD169. Moreover, the population of non-classical monocytes expressing CD169 increased with the percentage of monocyte subpopulations expressing TLR9. Similarly, the percentage of classical monocytes expressing TLR4 increased with the percentage of classical monocytes expressing CD209 and of intermediate monocytes expressing CD169, demonstrating the complexity of interactions within the monocyte compartment in deceased patients from the beginning of the infection. Moreover, intermediate monocytes expressing CD169 were also accompanied by increased expression of TLR4, and then TLR3, in the same subpopulation (**Figure 5C**).

Collectively, the analysis of these correlations suggests that early dysregulation of monocyte subset interactions and receptor expression may contribute to severe disease progression, underscoring the potential of these markers to inform prognosis and guide therapeutic strategies in patients with COVID-19.

## Discussion

COVID-19 struck the world as a pandemic caused by the SARS-CoV-2 virus, triggering an unprecedented global effort to develop effective vaccines to reduce morbidity and mortality (33). However, accumulating evidence indicates that immune protection induced by infection or vaccination can decline over time (34). Studies have shown that antibody and cellular immune responses can decrease within months after vaccination, supporting the need for booster doses to maintain protection (35).

Therefore, a deeper understanding of the immune system response during SARS-CoV-2 infection remains crucial for improving long-term protection and vaccine strategies.

Between July 2020 and March 2021, we had the opportunity to follow a cohort of unvaccinated COVID-19 patients in Belo Horizonte, Brazil, from week 1 of hospitalization until their clinical outcome. Here, we showed the results of monocyte subpopulations during the first week of hospitalization, comparing the groups according to outcome: discharged and deceased. It is important to mention that the patients presented at different times after the onset of their first COVID-19 symptoms, but only RT-qPCR-positive patients were recruited and included in this study, and all recruited patients were unvaccinated against SARS-CoV-2. We considered the first week of hospitalization a relevant time point, considering the worsening of symptoms that prompted patients to seek hospitalization. Moreover, this time point may allow the identification of parameters that could potentially be used as prognostic biomarkers.

Our study aimed to contribute to the characterization of monocytes as prognostic biomarkers during the acute phase of SARS-CoV-2 infection. Monocytes are an important immune system component in SARS-CoV-2 infection, and studies have demonstrated that they play a central role in the pathogenicity of the disease (14, 15). They are responsible for presenting antigens via MHC class II; however, if infected, the antigens could also be presented via MHC class I (36). These cell populations also have subclassifications that determine their migration to infected tissues and allow them to produce soluble components important to the immune response (37–39). We found that patients who had died had a lower percentage of classical monocytes and a higher percentage of non-classical monocytes. A recent study comparing COVID-19 patients with healthy individuals demonstrated an imbalance in monocyte subsets associated with disease severity and unfavorable outcomes (40). Our results are consistent with previous studies showing that depletion of classical monocytes is associated with severe cases of COVID-19, particularly in patients with diabetes mellitus (41). In contrast, non-classical monocytes have been linked to enhanced inflammatory responses and disease progression, with evidence of their migration to the lungs in severe cases (42).

However, the increased proportion of circulating non-classical monocytes observed in our study contrasts with previous reports showing reduced levels of these cells in the peripheral blood of patients with more severe disease (43). Importantly, this discrepancy may be explained by differences in study design, as our analysis focused on the first week of hospitalization and its association with clinical outcomes, whereas the cohort evaluated by Haschka and collaborators (43) included patients at varying stages of disease progression. Our findings, together with those of previous studies, suggest that the increased proportion of circulating non-classical monocytes during the early stages of the disease may contribute to their subsequent migration to infected tissues, promoting inflammation and tissue damage, particularly in the lungs, during later stages of COVID-19. This process may ultimately be associated with worse clinical outcomes.

After demonstrating that non-classical monocytes were more abundant in deceased patients compared with discharged patients during the first week of hospitalization, we next investigated the expression of CD169 in monocyte populations, along with CD209, a receptor associated with increased production of pro-inflammatory cytokines during SARS-CoV-2 infection. Overall, monocytes from deceased patients exhibited higher expression of both CD169 and CD209 compared with those from discharged patients. CD169 was more frequently expressed in non-classical monocytes from deceased patients than in those from discharged patients. Moreover, this population also exhibited higher expression levels of these receptors per cell during the first week of hospitalization. Notably, CD169 (Siglec-1) is a type I interferon–inducible receptor expressed on monocytes that is upregulated during viral infections, including SARS-CoV-2 infection, reflecting the activation of antiviral immune pathways (15, 44). Indeed, CD169 expression has been previously associated with disease severity and mortality in patients with COVID-19 (14, 15, 44). However, to our knowledge, this is the first study demonstrating its increased expression specifically in the non-classical monocyte subset during the first week of hospitalization. In contrast, although no significant differences were observed in the percentage of CD209-expressing monocytes in our study, the expression intensity of CD209 was markedly increased in intermediate and non-classical monocytes from deceased patients. CD209 is a C-type lectin receptor involved in pathogen recognition and antigen uptake and has been shown to facilitate interactions with a wide range of pathogens, including viruses (45, 46).

In particular, the higher frequency and expression intensity of CD169 in non-classical monocytes suggests that this subset may play an important role in the inflammatory response associated with severe COVID-19. Although the proportion of CD209-positive monocytes did not differ between groups, the increased expression levels observed in intermediate and non-classical monocytes in deceased patients further support the association of these receptors with disease severity and poor clinical outcomes. Overall, these results highlight the potential relevance of CD169- and CD209-expressing monocyte subsets as immunological markers associated with severe SARS-CoV-2 infection.

Other important molecules expressed by monocytes that have been reported to play a role in COVID-19 are Toll-like receptors. Our findings indicate that, early during COVID-19 hospitalization, deceased patients exhibit higher frequencies of TLR2-expressing monocytes across all subsets, along with increased TLR4 expression specifically in non-classical monocytes. Consistent with our findings, it has been reported that TLR2 directly recognizes the SARS-CoV-2 envelope (E) protein, contributing to the induction of inflammatory cytokine production and thereby promoting disease severity (28). In addition, TLR4 has been shown to interact with the SARS-CoV-2 spike protein, facilitating viral entry, upregulating ACE2 expression, and contributing to myocardial inflammation (26, 27). Together, these results suggest that dysregulated TLR2 and TLR4 expression in distinct monocyte subsets may contribute to disease severity, highlighting the potential of these receptors as early immunological markers for prognosis in patients with COVID-19 during the first week of hospitalization. Furthermore, the absence of significant differences in TLR3 and TLR9 expression underscores the selective involvement of TLR2 and TLR4 in the early immune response associated with severe outcomes.

Previously, we demonstrated that patients with increased expression of CD169, CD209, TLR2, and TLR4 in monocytes during the first week of hospitalization had worse outcomes. Building on these findings, we analyzed correlation networks between monocyte subsets and marker expression. Discharged patients exhibited more coordinated and homogeneous interactions, primarily within the same monocyte subsets, suggesting a more regulated immune response. In contrast, deceased patients showed more complex and heterogeneous networks, including negative correlations and cross-subset interactions, particularly involving CD169, TLR4, and TLR9. These results indicate that early dysregulation of monocyte subset interactions and receptor expression may contribute to disease severity. Importantly, the distinct correlation patterns observed between discharged and deceased patients suggest that not only the magnitude but also the organization of the immune response plays a critical role in disease progression. While survivors exhibited a more coordinated and regulated monocyte response, deceased patients showed a dysregulated and heterogeneous network, indicative of impaired immune control. The search for biomarkers and the characterization of all the pathophysiological processes involved in COVID-19, considered to be the largest pandemic of modern times, remain critical for elucidating disease progression and improving clinical outcomes (47–49). There is broad consensus among the scientific community and healthcare professionals that the assessment of routine laboratory biomarkers represents a fundamental step in the diagnosis and prognosis of COVID-19 (50, 51). However, it is essential to investigate factors that may influence or alter the expression of these biomarkers, including the effects of therapeutic interventions and immune modulation (51–54).

It is important to note that the samples analyzed in this study were collected during the first year of the pandemic, when no vaccines were available, and only a limited number of SARS-CoV-2 variants were circulating. Studies such as this one can help identify immune phenotypes associated with disease progression, thereby informing the development of immunobiological and therapeutic strategies aimed at preventing unfavorable clinical outcomes.

In conclusion, our findings demonstrate that early alterations in monocyte subset distribution and activation status are strongly associated with clinical outcomes in patients with COVID-19. Specifically, a reduced frequency of classical monocytes, coupled with an expansion of non-classical monocytes during the first week of hospitalization, was associated with mortality. Furthermore, increased expression of CD169, CD209, TLR2, and TLR4, particularly within non-classical and intermediate monocyte subsets, indicates a state of heightened innate immune activation that may contribute to disease severity. Together, these results support the concept that early monocyte-driven immune dysregulation is a key determinant of severe outcomes in COVID-19. Importantly, the identification of specific monocyte subsets and their associated markers as potential prognostic indicators provides valuable insights into disease pathogenesis and may contribute to the development of targeted therapeutic approaches and improved risk stratification in hospitalized patients.

## Materials and methods

### Cohort and ethics

This study was conducted in Belo Horizonte, Brazil. Before patient recruitment, ethical committee approval was obtained from the relevant institutions (CAAE 30399620.0.0000.5091; approval number 3.946.009). Between 21 July 2020 and 20 March 2021, hospitalized COVID-19 patients (n = 184) who tested positive for SARS-CoV-2 by swab and qPCR were enrolled in the study for blood collection during hospitalization, as shown in **Figure 6**. Exclusion criteria were a cause of hospitalization other than COVID-19, age below 18 years, and pregnancy (55). It is important to mention that during the study period, no COVID-19 vaccines were available in Brazil. Hence, all patients in this study were unvaccinated. Patients were distributed according to their hospitalization outcome: discharged (n = 142) and deceased (n = 42), regardless of the duration of hospitalization.

**Figure 6:**
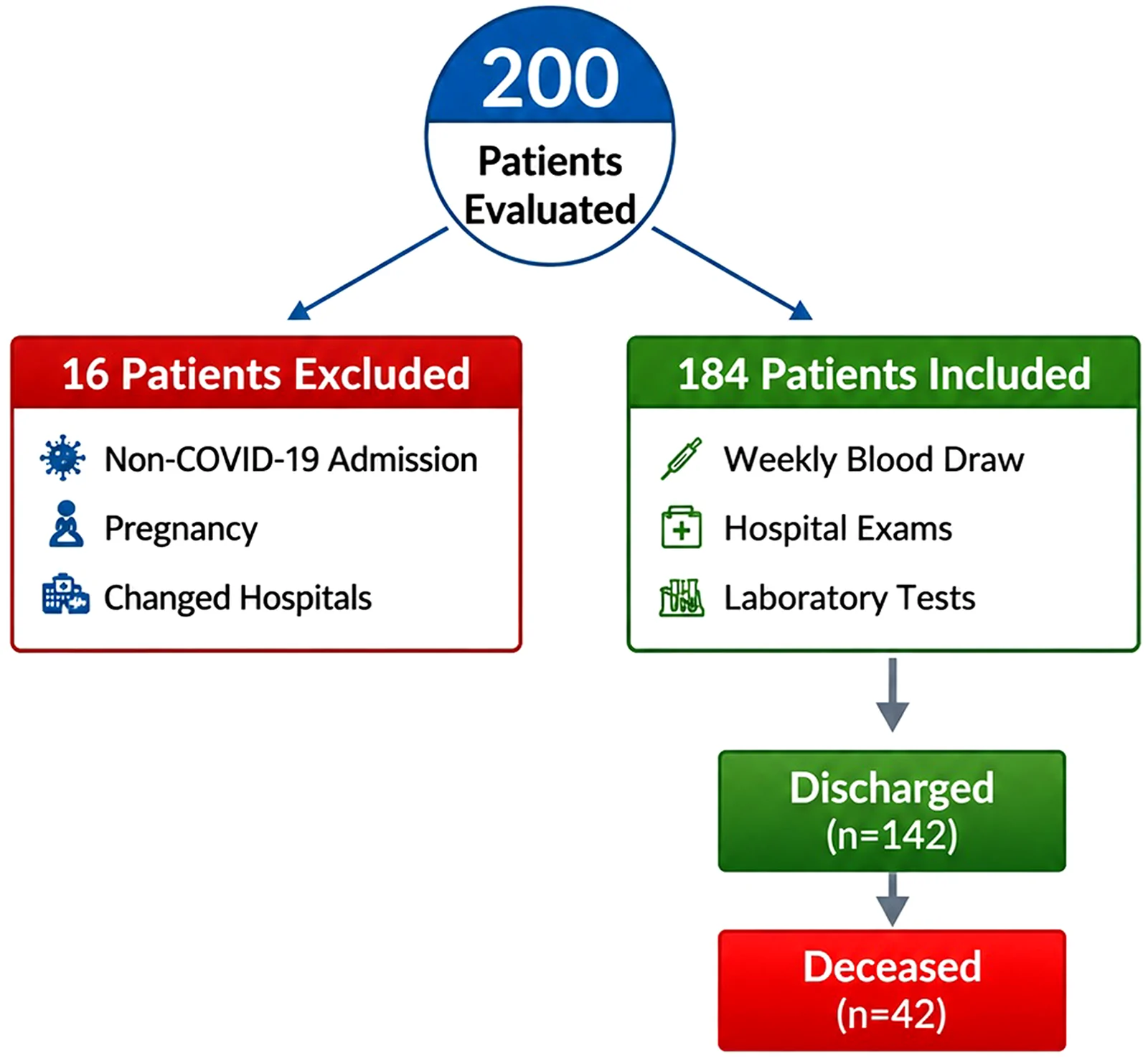
Study design and patient enrollment. Between 21 July 2020 and 20 March 2021, a total of 184 hospitalized patients with confirmed COVID-19 were enrolled in the study. Diagnosis was established via nasopharyngeal swab followed by quantitative PCR (qPCR) detection of SARS-CoV-2. Peripheral blood samples were collected during hospitalization for subsequent immunological analyses. The figure illustrates the timeline of patient inclusion and sample collection throughout the study period.

### Blood samples

All patients had 10mL of peripheral blood collected in a heparin tube during the first week of hospitalization. After collection, blood was processed in the laboratory by centrifugation at 300 g for 10min at 4°C. Plasma was frozen at –80°C immediately. The remaining cells received BD lysis buffer (BD Biosciences (San Jose, CA, USA), New Jersey, USA) according to the manufacturer’s instructions. Subsequently, the cells were counted using an automated Countess 2 (Thermo Fisher Scientific (Waltham, MA, USA), Massachusetts, EUA) and slowly frozen in 90% fetal bovine serum (FBS) and 10% DMSO. The cells were kept in liquid nitrogen until use. On the day of use, the cells were quickly thawed, transferred to 15 mL of collagenase/DNAse solution, washed three times in complete DMEM (10% FBS and 1% penicillin/streptomycin) (Thermo Fisher, Massachusetts, EUA), counted, and plated for flow cytometric analysis.

### Flow cytometric measurement

The cells were incubated with brefeldin A (10 µg/mL) for 4 h. The cells were then stained with the following surface markers, all of which were purchased from Invitrogen (Carlsbad, CA, USA): a) HLA-DR PE-Texas Red (clone TU36), b) CD14 Super Bright 645 (clone 61D3), c) CD16 APC eFluor 780 (clone CB16), d) CD169 Super Bright 436 (clone 7-239), e) CD209 PECy7 (clone eB-h209), f) TLR2 Alexa Fluor 488 (clone TL2.1), g) TLR4 Super Bright 600 (clone HTA125), and h) TLR9 APC (clone eB72-1665), along with the Live/Dead Fixable Aqua Dead Cell Stain Kit for 405 nm excitation. The cells were then fixed, permeabilized, and intracellularly stained for the following cytokines: a) IL-4 (clone 11B11), b) IFN-γ (clone XMG1.2), c) IL-5 (clone TRFK5), d) IL-17A (clone TC11-18H10.1), and e) IL-10 (clone JES5–16E3), all labeled with phycoerythrin (PE). For each sample, at least 100,000 cells were analyzed. The data were analyzed using FlowJo software and a FACS Fortessa flow cytometer (both from Becton Dickinson (Franklin Lakes, NJ, USA, San Jose, CA). The analysis strategy can be observed in **Supplementary Figure 1**.

### Statistical analysis

Flow cytometry data were analyzed using GraphPad Prism 10. A two-tailed non-parametric Mann–Whitney test was used to compare the discharged and deceased groups. Statistical significance was defined as a p-value < 0.05. For correlation comparison, a non-parametric Spearman test was performed, and the value of r was considered statistically relevant if > 0.7 for positive correlations (blue) and < –0.7 for negative correlations (red).

## Data Availability

The raw data supporting the conclusions of this article will be made available by the authors, without undue reservation.

## Supplementary material

Supplementary Figure 1 Flow cytometry gating strategy for monocyte subset identification and marker expression. Peripheral blood monocytes were analysed by flow cytometry following a sequential gating strategy. Singlets were first selected to exclude doublets, followed by gating on viable cells. Monocytes were then identified based on forward and side scatter properties in combination with HLA-DR expression. Monocyte subpopulations were defined according to CD14 and CD16 expression as classical (CD14brightCD16⁻; panel A), intermediate (CD14brightCD16⁺; panel B), and non-classical (CD14⁻CD16⁺; panel C). Representative histograms illustrate the expression of activation and pattern recognition markers, including CD169, CD209, TLR2, TLR4, and TLR9 within the monocyte population.

Supplementary Figure 2 CD169 and CD209 expression by monocytes subsets. Peripheral blood monocytes from hospitalized COVID-19 patients were analysed during the first week of hospitalization to assess the expression of CD169 and CD209, deceased and dischargd patients. Percentage of expression of CD169 by monocytes **(A–C)** and MFI **(D, E)**. Percentage of expression of CD209 by monocytes **(F–H)** and MFI **(I–L)**. The statistical values are presented in the graph.

Supplementary Figure 3 TLR2 and TLR4 expression by monocytes subsets. Peripheral blood monocytes from hospitalized COVID-19 patients were analysed during the first week of hospitalization to assess the expression of TLR2 and TLR4, deceased and dischargd patients. Percentage of expression of TLR2 by monocytes **(A–C)** and MFI **(D, E)**. Percentage of expression of TLR4 by monocytes **(F–H)** and MFI **(I–L)**. The statistical values are presented in the graph.
